# Scots Pine (*Pinus sylvestris* L.) Growth Suppression and Adverse Effects on Human Health Due to Air Pollution in the Upper Silesian Industrial District (USID), Southern Poland

**DOI:** 10.1007/s11270-012-1114-8

**Published:** 2012-03-01

**Authors:** Ireneusz Malik, Małgorzata Danek, Ewa Marchwińska-Wyrwał, Tomasz Danek, Małgorzata Wistuba, Marek Krąpiec

**Affiliations:** 1 Faculty of Earth Sciences, University of Silesia, Będzińska 60, 41-200 Sosnowiec, Poland; 2 Faculty of Geology, Geophysics and Environmental Protection, AGH-University of Science and Technology, al. Mickiewicza 30, 30-059 Krakow, Poland; 3 Department of Environmental Health, Medical University of Silesia, Piekarska 18, 41-902 Bytom, Poland

**Keywords:** Air pollution, Tree ring reductions, Infant mortality, Cancer morbidity

## Abstract

Air pollution emissions were not continually monitored in the Upper Silesian Industrial District (USID), southern Poland, and data is only available for the last 20 years. Long-lasting and severe tree ring reductions in pines growing 5–20 km north of the USID area recorded particularly high levels of air pollution emissions in the period 1950–1990. Especially high amounts of reductions and many missing rings were found in the period 1964–1981. At the same time, pines growing 60 km west of the USID do not record deep ring reductions; this proves that the phenomenon is of a regional nature. Increases in infant mortality and lung, bronchial, and tracheal cancer morbidity rates among males were also recorded in the USID during periods of high air pollution. Infant mortality rates increased several years after the tree ring reductions. Therefore, it may be possible to use tree ring reductions as an early indicator of the occurrence of adverse effects on human health.

## Introduction

Emissions of SO_2_, NO_x_, and other phytotoxic compounds lead to serious disturbances in tree physiology and metabolism (Farrar et al. 1977), which are in turn reflected by a decrease in the width of annual tree rings. Analyses of tree ring width reduction as a result of atmospheric pollution date back to the second half of the nineteenth century (Stoeckhardt 1871). Many studies were carried out in the second half of the twentieth century when intensification of air pollution led to the degradation of increasingly large areas of forests, especially in the 1970s. The studies were conducted near different sources of pollution (Ashby and Fritts 1972; Vinš and Mrkva 1973). It was demonstrated that the relationship between tree ring width and climate was different in trees growing near sources of pollution (Nash et al. 1975; Juknys et al. 2003). The studies of Vinš and Mrkva (1973), Thompson (1981), Kennedy-Sutherland and Martin (1990), Nöjd et al. (1996), Juknys et al. 2003, and Elling et al. (2009) revealed different intensities of radial tree growth reduction depending on their distance from the pollution source and the amount of pollution emitted into the atmosphere.

In the last 20 years, studies have often been carried out near individual sources of pollution: at a non-ferrous smelter (Nöjd et al. 1996), a chemical plant (Jämbäck et al. 1999), a metal extraction and processing plant (Ivshin and Shiytov 1995), and a copper smelter (Kennedy-Sutherland and Martin 1990).

A study carried out by Schweingruber et al. (1985) applied a new method based on the analysis of pointer years and abrupt growth release. The study showed the number, degree, and spatial and temporal distribution of tree ring reductions. It was found that both a deterioration and an improvement in environmental conditions can be observed in tree rings a few years before they can be detected by the assessment of the condition of the tree crown (Schweingruber et al. 1985; Kontic and Winkler-Seifert 1987).

Studies concerning the impact of air pollution on annual tree ring widths have been conducted in Poland in the Niepołomice Forest, Ojców National Park (Krąpiec and Szychowska-Krąpiec 2001), near the Nitrogen Fertilizer Plant in Puławy (Oleksyn 1988) and close to the Police Chemical Plant (Szychowska-Krapiec and Wiśniewski 1996). A general intensification of the suppression of tree growth was observed in the eastern part of the Upper Silesian Industrial District (USID) between 1960 and 1990, as well as a noticeable correlation between this and the level and duration of emissions (Danek 2007).

Atmospheric pollution also leads to an increase in human disease and mortality rates. The epidemiological evidence suggests that adverse health effects depend both on the concentrations and durations of exposure.

Long-term exposure has a larger, more persistent, cumulative effect than short-term exposure (Pope 2007). According to the WHO Global Burden of Disease Comparative Risk Assessment (Cohen et al. 2005), air pollution is associated with a wide range of acute and chronic health effects, the nature of which may vary depending on the pollutant constituents. Outdoor air pollution is estimated to be responsible for about 5% of tracheal, bronchial, and lung cancer mortalities, and about 1% of child mortalities from acute respiratory infections in urban areas worldwide (The European Health Report, 2009). This statistic only concerns mortality rates; if cardiopulmonary disease rate data is also taken into account, the proportion grows to 20% (Cohen et al. 2005).

A study carried out in São Paulo shows that the effect of air pollution on mortality is a result of variations in SO_2_ concentrations. Higher outdoor particulates concentrations are associated with increased cardiopulmonary death rates in cohort studies (Hoek et al. 2002) and there is a stable, negative association between air pollution and birth weight (Bell et al. 2007; Gray et al. 2010). Studies have shown that air pollution may increase the risk of adverse birth outcomes, for example the risk of premature birth, by 25% per 10 μg/m^3^ increase in NO_2_ concentrations (Maroziene and Grazuleviciene 2002).

The current study of the concentration of airborne particulate and gaseous pollutants in the Upper Silesian Industrial District (USID) has proved the influence of these pollutants on the daily mortality pattern of the inhabitants (Kowalska et al. 2007).

Previous and present-day studies suggest that SO_2_ remains the most significant determinant among the air pollutants examined (Zejda 2000; Kowalska et al. 2010). (Marchwinska-Wyrwał et al. 2010) have used the Silesian District as a case study to look at the potential health consequences of chronic exposure to air pollution. This case study demonstrated the relationship between air pollution and the cause of some diseases. Absalon and Ślesak (2010) suggest the existence of a relationship between cadmium–lead air pollution and cancer among children, even in the period 1995–2007, when harmful emissions in the USID were relatively low.

Only discontinuous and selective air pollution data is available from communist times (between the Second World War and 1989) in the USID. Due to the existence of a relationship between the level of air pollution and losses observed in the annual increment of trees, and as the distribution of the reduction in tree rings can reflect the distribution of pollution in time, in this study ring reductions in trees growing around the USID are used as indicator of the level of air pollution. Due to this, it is possible to compare the amount of pollution recorded in tree ring reductions with the human disease rates and mortality data.

The general objective of the study is to determine the impact of the air pollution level on pine tree growth and human health within the USID. The specific objectives are:

1. Reconstruction of air pollution emissions in the last 80 years, determined using the growth suppression of pines to the north of the USID, in particular:
  - To develop site and local chronologies (for pines growing about 5–20 km from the USID) and a reference chronology (for pines growing 60 km from the USID),
  - To determine tree ring reductions and missing rings in pines growing 5–20 km from the USID,
  - To compare periods with tree ring reductions and air pollution in the whole USID (determined indirectly by the level of coal exploitation, electric energy, and steel production) and local emitters (SO_2_ emissions from the Miasteczko Śląskie Zinc Foundry, production volume at the Tarnowskie Góry Chemical Plant).
2. Comparison of the variability of ring reductions in pines with infant mortality and the lung, bronchial, and tracheal cancer morbidity rates among the male population in the USID.
3. Assessment of the possibility of using tree ring reductions as an indicator of adverse effects on human health.

## Study Area

### General Information

**Fig. 1:**
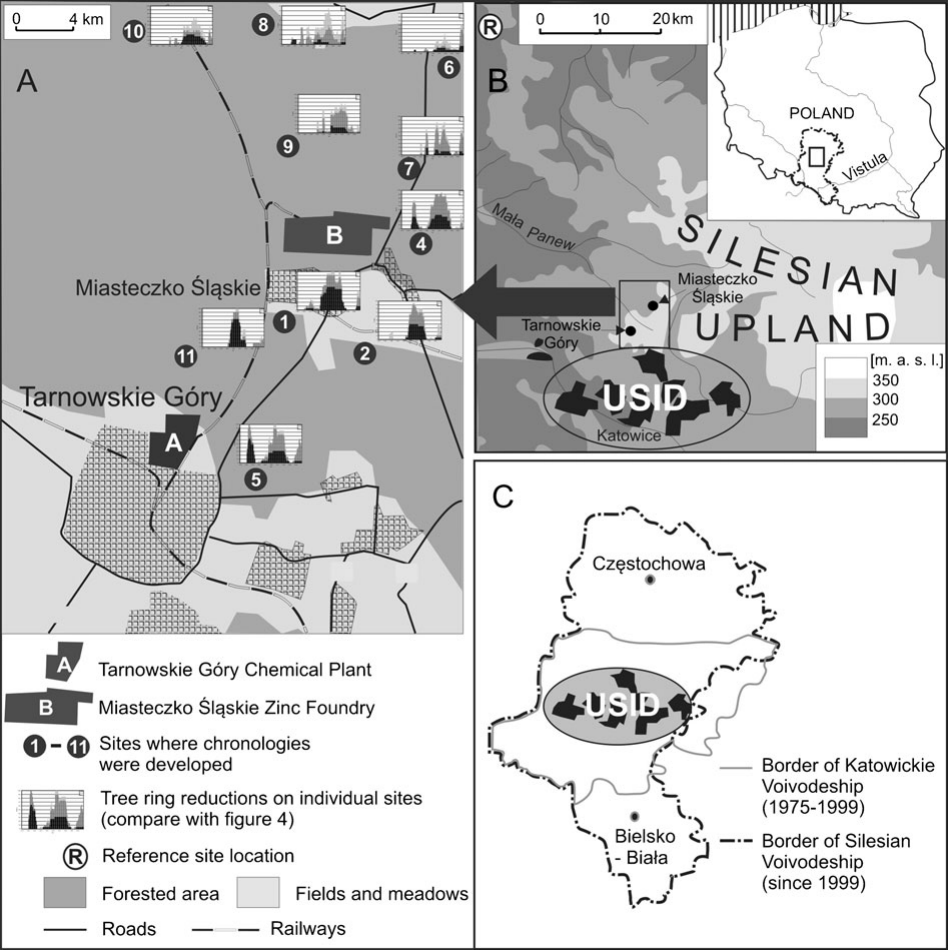
Location of the study area. **a** Study sites in the vicinity of Tarnowskie Góry town with tree ring reductions graphs. **b** Study area in Poland and Silesian Upland. **c** Upper Silesian Industrial District site in the Katowickie/Silesian Voivodeship

The study was carried out in the Silesian Upland, about 5–20 km north of the USID, in the Silesian Voivodeship, where heavy industry operated without any environmental protection until 1990 (Fig. 1a–c). Sampling sites for dendrochronological studies were located in a flat area composed of sand and gravel where podzolic soils developed. Southwesterly and westerly winds dominate in the study area. Mean annual precipitation fluctuates at around 700 mm.

Ten sampling sites were selected relatively close to the Tarnowskie Góry Chemical Plant (sites 5 and 11—4 km; sites 1 and 2—9 km) and the Miasteczko Śląskie Zinc Foundry (sites 1, 2, 4, 7, and 9—from 3 to 5 km; sites 6, 8, and 10—from 9 to 12 km) (Fig. 1a–b). Within the USID, it was not possible to find a forest similar in age and composed of one species. Therefore, sampling sites for dendrochronological study were selected as close as possible to the USID, where planted pine monocultures were growing. In the study area, dry-mesic pine forest (36%) and humid pine forest (25%) dominate. Eighty-two percent of trees in the study area are Scots Pines (*Pinus sylvestris* L.) (Wójcik and Buczkowski 2001).

### History of Heavy Industry in the Upper Silesian Industrial District (USID)

Coal-based heavy industry dates back to the eighteenth century in the USID. In 1796, the first iron smelter heated with coke was built and the first steam engine was brought to the region. Since then, coal exploitation, the iron and steel industry, lead and zinc smelting, the chemical industry, and different branches of the processing industry have flourished in the Upper Silesia Industrial District. This quick development was disrupted by the damage caused by the First and the Second World Wars. Prior to the Second World War, the peaks of industrial prosperity occurred at the beginning of the twentieth century and in the late 1930s.

Data related to coal exploitation and steel production was chosen to illustrate the post-war industrial growth in the USID (Statistical Yearbooks 1958–2009). The reasons for this were a lack of air pollution data for the USID during communism. The data mentioned was chosen because it contained the longest available time sequences and the most complete data.

Coal and steel were also the basis of almost all industrial production in the USID—indirectly they indicate the course of development of the whole region. Industrial production data in the period between the end of the Second World War and 1989 was subject to communist propaganda and could have been manipulated for political purposes. This especially applies to coal exploitation and steel production. Because of this, a third indicator—electrical energy production—was added, as it was less interesting from a propaganda perspective. Electrical energy is also necessary for production processes in the majority of more advanced industrial plants. In addition, before 1989 the consumption of electrical energy by individual users (households) was very low. Thus, the rate of electricity production is a good indicator of the development of the processing industry in the USID.

**Fig. 2:**
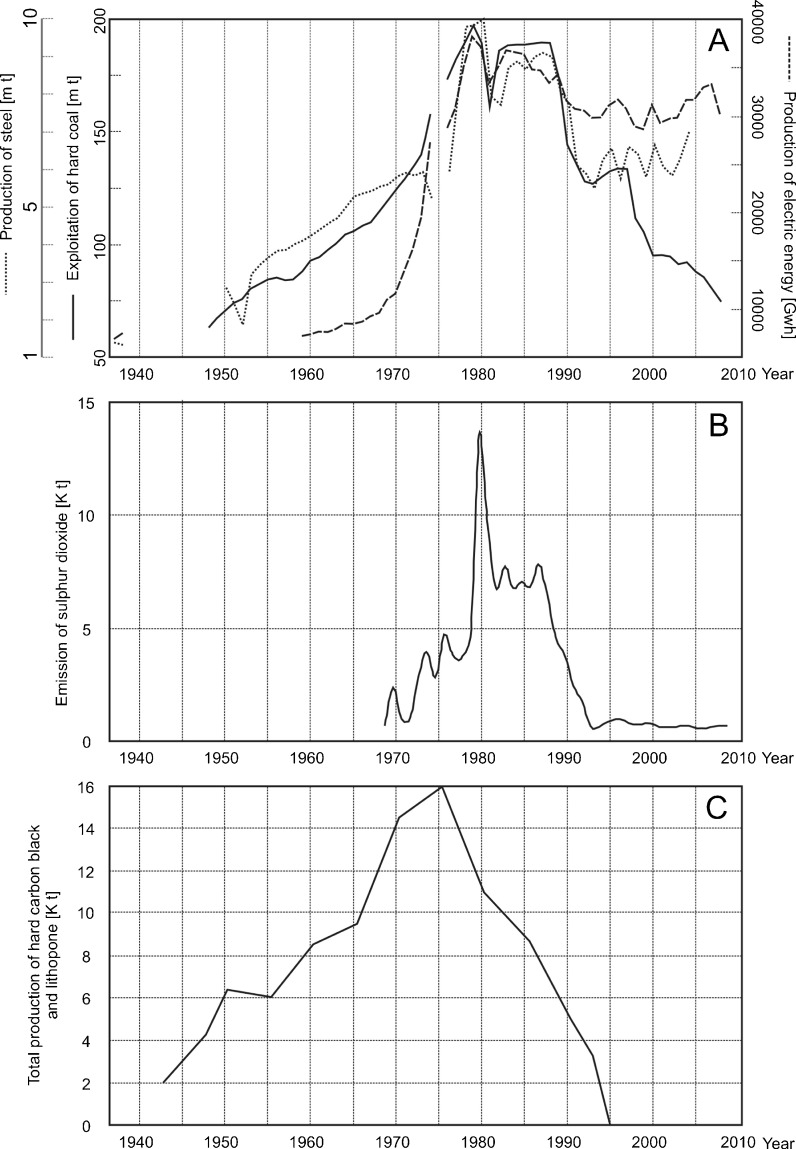
Indicators of industrial production and pollution in the USID in the period 1937–2010. **a** The Katowickie/Silesian Voivodeship. **b** The Miasteczko Śląskie zinc smelter. **c** The chemical plant in Tarnowskie Góry

After the Second World War, heavy industry in the region was organized to suit communist conditions, those of the centrally planned economy of the Polish People’s Republic with its requirements of constant growth. The increase in industrial production rates began midway through the 1960s, with a peak in the late 1970s and 1980s (exploitation of coal, production of steel and electrical energy—Fig. 2a). In the late 1970s, production was four (in the case of coal), five (steel) to eight (electrical energy) times higher than in the 1960s (20–30 years earlier, see Fig. 2a).

The decline of heavy industry in the Upper Silesian Industrial District began at the end of 1980s (Fig. 2a) with the crisis of the communist planned economy and the beginning of a democratic and economic transformation. Many coal mines, steelworks, and other factories were closed due to their unprofitability. This was also a period in which ecological consciousness grew. Standards of environment protection were established and applied to industrial plants, which often led to a decrease in production. In the first half of the 1990s, the production of coal and steel was almost half than in the late 1970s (15 years earlier, see Fig. 2a).

In the last 10–15 years, we have observed the return of heavy industry to the USID. The production of steel and electric energy is growing (Fig. 2) and more advanced branches of the processing industry are also growing slightly.

### History of the Tarnowskie Góry Chemical Plant

The Tarnowskie Góry Chemical Plant had been operating since 1922 at the site of a previous iron foundry and paperwork. The chemical plant entered into liquidation in 1995. The very serious pollution to the environment detected close to chemical plant meant that plant was included in the list of 18 biggest polluters in Poland in 1994. The chemical plant produced organic chemical compounds, in particular black carbon, at the beginning of its operation. The production of inorganic compounds increased gradually since 1965 and was focused primarily on lithopone (Biernacki 1983). The production of both substances was harmful to the environment. During the production of black carbon, corrosive clouds of anthracene and naftalene were emitted into atmosphere and affected the nearby pine forest. In the 1960s, due to these emissions, all pines growing within a 1-km radius of the chemical plant were already dead. As a result of lithopone production, large amounts of sulfur dioxide and hydrogen chloride were emitted into atmosphere and also destroyed a nearby pine forest. The production of black carbon was at its greatest in 1955–1970 and the production of lithopone in 1965–1985 (Malik et al. 2010) (Fig. 2b).

### History of the Miasteczko Śląskie Zinc Foundry

The Miasteczko Śląskie Zinc Foundry has been operating since 1961. At its beginning, the plant mainly produced zinc, lead, and sulfuric acid. The plant’s technological processes caused harmful atmospheric emissions, especially of SO_2_. The foundry history can be classified into three periods. The first is the foundry expansion between 1961 and 1972; the second period, between 1972 and 1989, is characterized by an increase in zinc and lead production, and a connected increase of harmful atmosphere emissions. The third period started in the 1990s, when environment-friendly technology was developed in the foundry. As a consequence, the amount of pollution emitted, especially SO_2_, was reduced (Bojanowski 2008).

The amount of SO_2_ emitted into atmosphere by the foundry was changing in the last 50 years. In the 1960s, SO_2_ emissions were five to eight times greater than in the last few years (Fig. 2c). Specially large amounts of emissions were recorded in 1979 when new assembly lines were organized, at this time the level of emissions was 20 times greater than now. In the 1980s, the foundry released SO_2_ emissions two times greater than in the 1970s. Since 1991, emissions have been reduced considerably and remain at the same level today (Malik et al. 2009).

## Materials and Methods

### Statistical Data on Industrial Production and Environmental Pollution

In this study, the authors used data from the following: coal exploitation (1948–2008, gap in 1975), electrical energy (1959–2008, gap in 1975), and steel production (1950–2004, gap in 1975) in the Katowickie/Silesian Voivodeship. It was assumed that the variability of coal exploitation, and steel and electricity production reflected the relative changes in air pollution emissions in the USID.

The administrative and territorial division of Poland, including Upper Silesia, has changed in the last 60 years (Fig. 1c). The cities of the USID comprise about 70% of the population of the former Katowickie Voivodeship (1975–1998) and about 40–50% of the population of the former and current Silesian Voivodeship. Despite this, industrial production data and epidemiological data were collected for the whole Katowickie/Silesian Voivodeship. This data is used in the context of the USID in the following part of the paper because of the dominance of the USID in the population and industry of the region. SO_2_ emission data for 1968–2008 was obtained from the Miasteczko Śląskie Zinc Foundry archives; data on other pollutants was not available but the variability of SO_2_ emissions changed simultaneously with the emissions of other pollutants emitted from the zinc foundry. Because of the closure of the Tarnowskie Góry Chemical Plant, it was only possible to obtain information about the production volumes; atmospheric emission data was not available. Increases in industrial production and SO_2_ emissions were accompanied by increases in the emission of other pollutants, like trace metals, soot particulates, aromatic hydrocarbons, and their derivatives, and carcinogenic compounds. In this study, we have assumed that total industrial air pollution is harmful both to trees and to humans and that it was not possible to indicate what was the role of individual pollutants in adverse effects on health.

### Dendrochronological Record of Scots Pine (*P. sylvestris* L.)

Because of the species composition of local forests in the study area, the tree taxon selected for sampling was also Scots pine. Coniferous trees (among them pines) were observed as those first disappearing in areas with high concentrations of sulfur dioxide in the air. Scots pine is considered a species sensitive to sulfur dioxide, more vulnerable than larch and less than fir and spruce (Greszta et al. 2002). Experimental studies were conducted on pine saplings subjected to the impact of acid rain. These studies have revealed that with an increase in acidity (from pH 5 to pH 2), the length of the previous year’s vertical growth decreased by a factor of 6 (Greszta et al. 2002).

There were 220 cores taken, using a Pressler borer, from pines growing near the chemical plant and zinc foundry at 11 sampling sites in November 2009. In this paper, we have only used results from 10 sites (200 cores). At one of the sites (no. 3), we found the cross-dating of tree ring series difficult because too many rings were missing. As a result, site 3 was eliminated. Twenty trees were sampled at each site (one core per tree). We had permission to take one core per tree, so we abandoned the standard procedure which recommended two sample cores per tree. Cores from 20 trees were also collected at a reference site located about 60 km northwest of the USID, which is located relatively far away from the harmful emission sources. At all sites, including the reference site, pines were growing in mixed fresh coniferous forests. Cores were taken from approximately 90-year-old pines at breast height. The cores were mounted in wooden holders and sanded with successively finer grades of sandpaper. Afterwards, the annual ring widths were measured with precision of up to 0.01 mm using the Dendrolab measuring system.

Tree ring series from all pines were fitted visually and correlated using TREE-RINGS and Quercus software (Walanus 2002). Based on the individual tree ring curves collected from pines growing close to the industrial plants in Miasteczko Śląskie and Tarnowskie Góry, site chronologies were developed as result of the mean ring width calculated for each year from all the trees growing at each individual site. Additionally, the local chronology for all trees growing close to the chemical plant and zinc foundry was determined as result of the mean ring width calculated for each year from all the collected cores. Standard chronologies were developed in ARSTAN software for the purpose of eliminating the fluctuation resulting from trees aging (Cook and Holmes 1999). When a chronology is developed, we need to eliminate age-related trends from individual tree ring series (usually of negative exponential or negative slope) to emphasize a common signal existing in all of them induced by the influence of climate, pollution, etc. In the first stage of the detrending algorithm, an exponential curve or straight line was fitted to every single tree ring series. The use of trend approximation (exponential or linear) depended on the shape of the observed data. In the next step, all the measured values were divided by the related value of a fitted trend. As a result, indexed dimensionless values without age trend were obtained.

In addition, the pointer years were also analyzed and the abrupt growth changes method was applied (Schweingruber et al. 1990). The ring reduction periods were determined for each tree ring curve. Ring reductions were found in case of a sudden decrease in the tree ring width. Reduction values were calculated as the total tree ring width of all rings in a reduction period in relation to the total tree ring width of all the same number of rings from the period preceding the reduction period (Schweingruber et al. 1985). Calculated reductions were classified into three groups—moderate reductions: 30–50%, strong reductions: 51–70%, and very strong reductions: >70%. Reductions weaker than 30% and shorter than 3 years were not taken into consideration. The Quercus software was used to determine reduction periods and calculate their values (Walanus 2002).

### Epidemiological Data

Epidemiological data was collected from the Statistical Bulletins of the Ministry of Health (1966–2009) and from the Polish Institute of Oncology. It was possible to collect relatively long data series of infant mortality (1965–2005) and lung, bronchial, and tracheal cancer morbidity rates for males (1970–2008, lack of data for the Silesian/Katowickie Voivodeship in 1974, 1981, and 1996–1998). Infant mortality data was reduced to prenatal mortality (duration of pregnancy disturbances, status of the fetus or infant resulting in maternal outcomes, intrauterine hypoxia, hemolytic disease of fetus and infant, birth trauma, additional reasons for prenatal mortality). In the case of cancer morbidity, we only took the male population into consideration because it was mainly males that worked in heavy industry and chemical plants, so the effect of any increase in male cancer morbidity should be more clearly visible than in the case of females. It was not possible to collect data for other diseases which was taken into account such as asthma and birth defects because the data series was uncompleted.

To find a possible correlation between tree ring reductions and infant mortality or lung, bronchial, and tracheal cancer morbidity rates in males, all the medical data (infant mortality per 10,000 life birth and lung, bronchus, and trachea cancers men morbidity per 100,000 people) was detrended and lagged by 1, 2, 3… up to 20 years. The detrending procedure was necessary because strong temporal trends related to the development in medical care in Poland (in the case of infant mortality) and the increase in morbidity rates recorded in Poland (in the case of cancer) were clearly evident. The index (detrended) rates were obtained by subtracting average rates of adverse human health effects for the whole of Poland from the rates in Upper Silesia (year by year). These relative parameters—the infant mortality index and cancer morbidity index—were used in further analyses. The detrending procedure allowed the elimination of population trends in the occurrence of adverse human health effects in the whole of Poland from the results. Detrending allowed the elimination of the influence of factors which are identical in the whole of Poland (for instance, tobacco smoking, UV-B radiation, etc.) from the results and interpretation. All correlations were lagged to test if tree ring reductions can be used as a kind of predictive instrument for the future health-related consequences of increasing pollution.

## Results and Discussion

### Comparison of the Local and Reference Chronologies

**Fig. 3:**
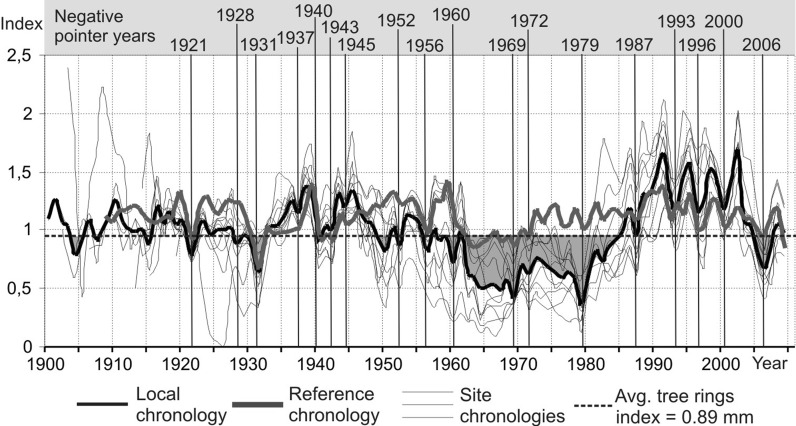
Standardized reference, local and site chronologies developed from tree ring series from sampled pines with negative pointer years marked. Values below the overall average tree ring index for all pines (0.89) are shaded *gray*

We developed a reference chronology for pines growing around 60 km from the Tarnowskie Góry Chemical Plant and the Miasteczko Śląskie Zinc Foundry (Fig. 3). A chronology was also developed for all pines sampled at individual sites located near both plants (local chronology) (Fig. 3). Negative pointer years (1921, 1928, 1931, 1937, 1940, 1943, 1945, 1952, 1956, 1960, 1969, 1971–1972, 1979, 1987, 1993, 1996, 2000, 2006) occur both in the local (developed near the plants) and reference chronology (Fig. 3). Because of this, the abovementioned narrow rings identified as pointer years are not considered to have been formed mainly due to the harmful atmosphere emissions from the Upper Silesian Industrial District or the two studied plants; e.g., tree ring reductions in 1940–1941 are usually clearly visible in rings of pines growing in Central Europe—they were formed due to the especially harsh winter of 1940 (Malik et al. 2009). In 1921, an extraordinary dry year could have been crucial to studied pines in forming extremely narrow annual rings (Zielski 1996). Also, other harsh winters, which occurred in Poland in 1956, 1960, 1962, 1976, 1979, and 1996, could have suppressed pine growth. Tree rings are particularly narrow when a harsh winter is followed by vegetation period with limited amounts of precipitation (Wilczyński and Skrzyszewski 2002).

Pines growing at study sites which comprised the local chronology generally produced narrow rings between 1960 and 1985 (Fig. 3). The tree ring index for this period is considerably lower than the average (0.89) for both the local chronology and all individual site chronologies (Fig. 3). At the same time (1960–1985), the trees which the reference chronology was composed of formed relatively wider annual rings (Fig. 3). This means that, in all probability, air pollution suppressed pine growth near the Upper Silesian Industrial District. The soil in the sites studied is undoubtedly strongly contaminated due to industrial activity in the region, but bad soil conditions did not play a significant part in the suppression of pine growth. After the harmful air emissions stopped in about 1990, pines started to produce tree rings that were remarkably wider (even wider than in reference sites), regardless of the continuing soil contamination. This suggests that despite the long residence time of elements in the soil, bad soil conditions do not control pine growth as much as air pollution.

Previous studies carried out at other locations near the USID also linked tree growth suppressions to air pollution (Danek 2007).

### Tree Ring Reductions and Missing Rings in Pines Growing near the USID

**Fig. 4:**
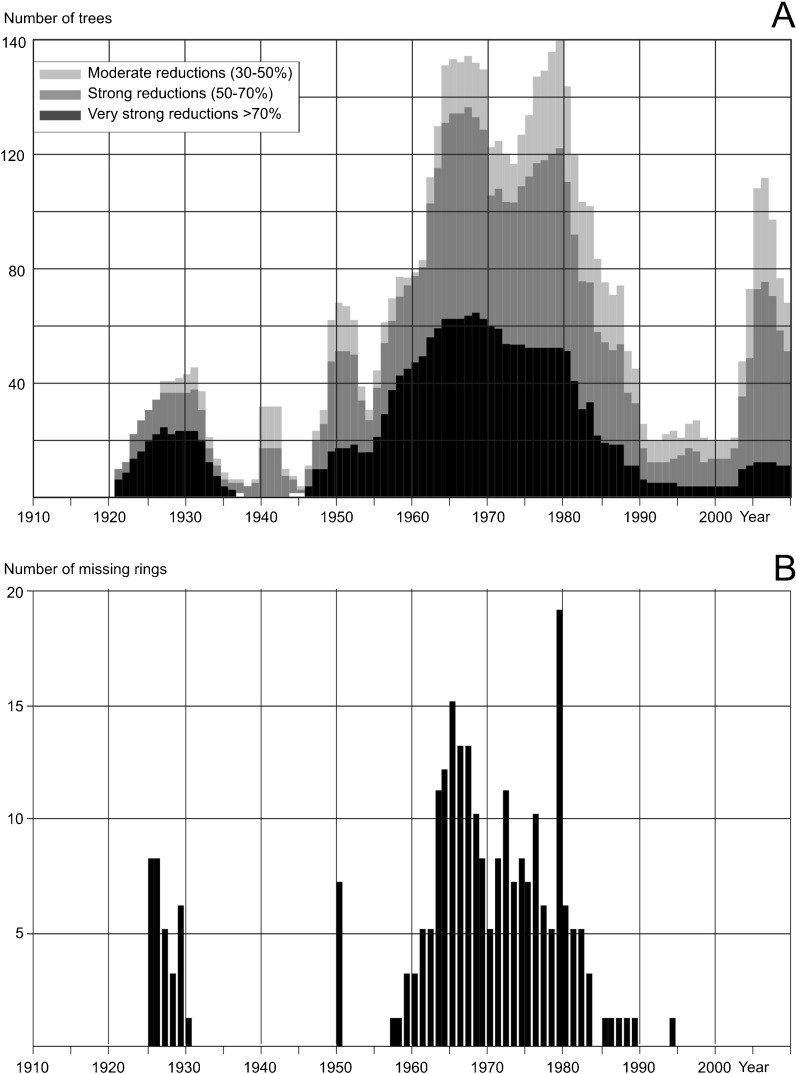
Graph showing the ring reductions detected in cores (**a**) and the number of missing rings identified in cores (**b**) collected from all pines sampled

**Fig. 5:**
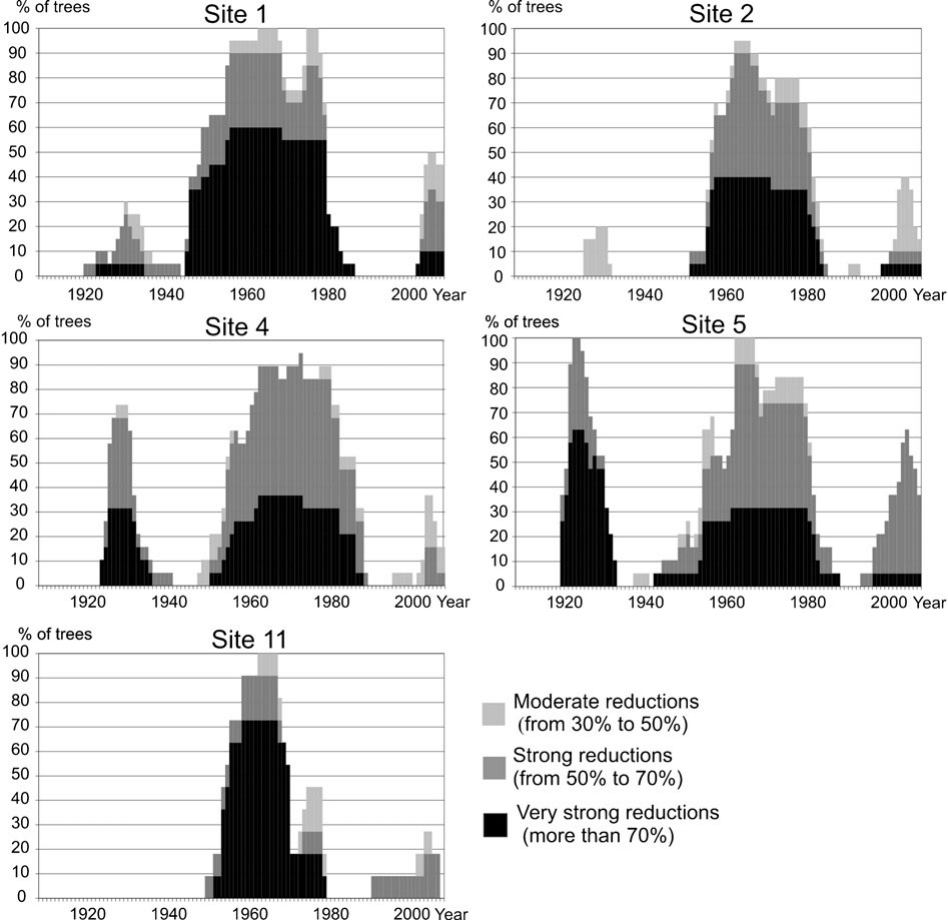
Graphs showing the pines ring reductions growing at individual sampling sites (nos. 1, 2, 4, 5, and 11)

**Fig. 6:**
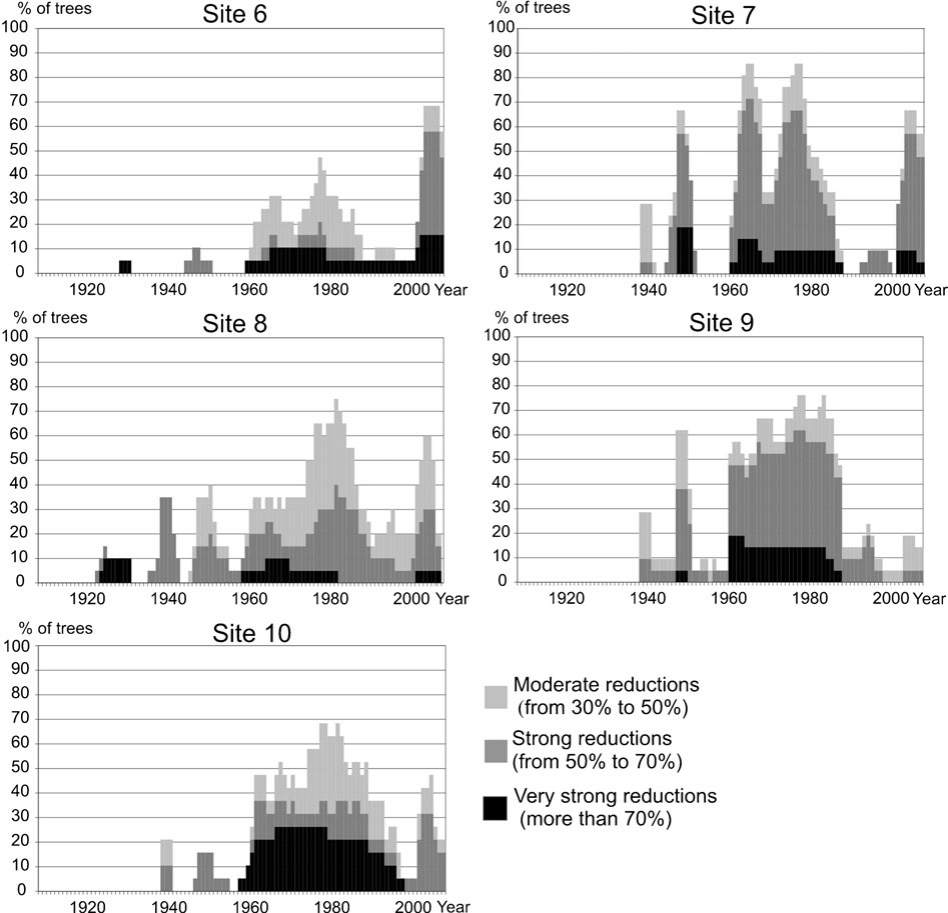
Graphs showing the pines ring reductions growing at individual sampling sites (nos. 6, 7, 8, 9, and 10)

Strong tree ring reductions were discovered in pines growing near the USID (Figs. 4a, 5, and 6). The first period which showed reductions was 1925–1935. In the sampled pines, reductions were also recorded between 1950 and 1990, and since 2003 until now. The highest number of tree ring reductions occurred from 1964 to 1981. This suggests that air pollution was at its highest level during mentioned periods. Many missing rings were found in obtained tree ring series and, in general, they occurred at the same time as the highest number of reductions (Fig. 4b). The first period with missing rings was from 1925 to 1930. The second occurred between 1957 and 1990. Two peak years with the highest number of missing rings were detected: 1964 and 1979 (Fig. 4b).

### Local and Regional Air Pollution as a Limitation of Tree Growth at the Study Sites

Pines growing directly next to the chemical plant and zinc foundry (sites 1, 2, 4, 5, and 11) (Fig. 5) had strongly reduced rings compared to the rings formed by pines growing further from both plants (sites 6, 7, 8, 9, and 10) (Fig. 6). The influence of local industry is normally evident in a large number of ring reductions, especially the most severe ones (Ivshin and Shiytov 1995). Because of this, the results obtained suggest that local emitters played an important role in the tree growth pattern in the studied area. However, it is also highly probable that the observed ring reductions were partially due to a pollution transfer from industry located in the whole of the Upper Silesian Industrial District. The reason for this is the simultaneous development of the USID and local industry in the studied area, confirmed by the gathered statistical data: coal exploitation, and steel and electricity production compared with production output and the emission of pollutants from chosen plants (Fig. 2).

**Fig. 7:**
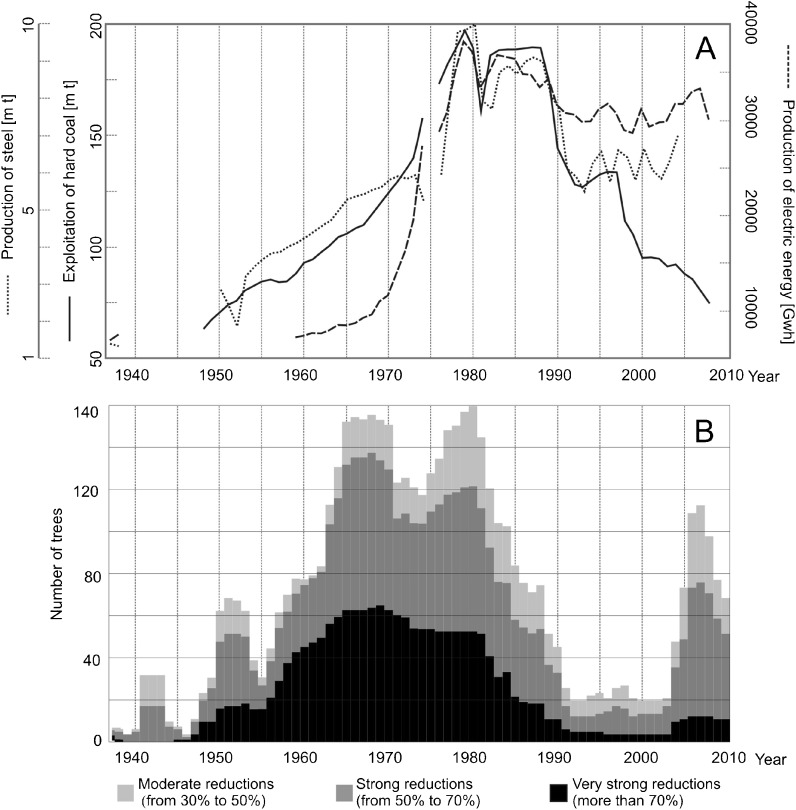
Comparison of coal exploitation, and steel and electrical energy production in the USID with tree ring index and tree ring reductions. **a** Comparison of production of steel, electric energy, and exploitation of hard coal in the USID. **b** Number of sampled pines with ring reductions

The transportation of pollution over long distances can contribute to the reductions in trees growing even several dozen kilometers from the emission sources (Danek 2007). In the studied area, it is not possible to separate regional and local effects on tree ring reductions. It is not clear what part of the observed reductions should be contributed to regional air pollution from the USID. As confirmation of not only the local but also probable regional checks of pine growth at the study sites, we observed a clear inverse ratio between the increase of coal exploitation, and steel and electrical energy production (Statistical Yearbooks of Katowice/Silesian Voivodeship 1958–2009), and the number of ring reductions (Fig. 7).

The relationship between air pollution emissions and the condition of the pines is also visible in a large number of missing rings, mostly in 1964–1981, when emissions of pollutants were especially high (Fig. 4b). In some cores, we found several missing rings one year after another. Such a large impact of pollution on tree growth has hardly ever been detected in Europe. Based on verbal information from the Forest Inspectorate that manages the studied forests, in the 1960s and 1970s the growth of trees in the area of interest seemed to stop completely. The tree ring width index started to increase after 1985; however, the total coal mined and steel and electricity production was high up to 1988. This lag seems to be an effect of the development of environmentally friendly technology in the USID in the second half of the 1980s.

### The Influence of Air Pollutants Emitted from the Miasteczko Śląskie Zinc Foundry on Pines Growth

**Fig. 8:**
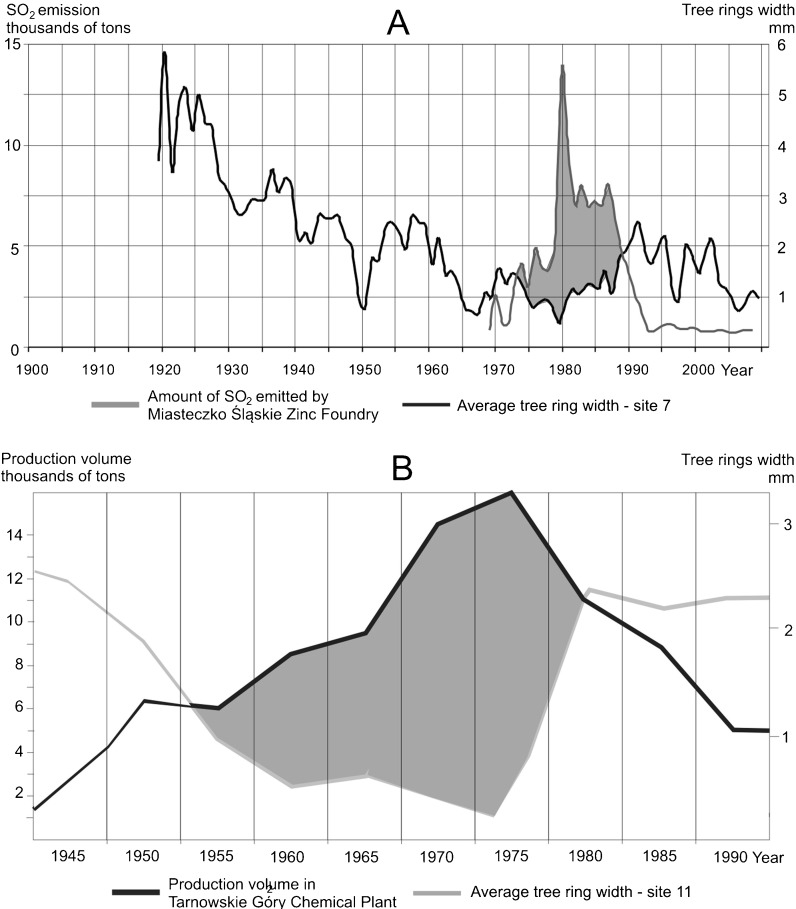
Graphs showing the relationship between tree ring widths, SO_2_ emissions, and the amount of industrial production at studied plants. **a** Inverse ratio between tree ring widths at site 7 and the level of SO_2_ emissions from the Miasteczko Śląskie Zinc Foundry. **b** Inverse ratio between the tree ring widths at site 11 and the level of production at the Tarnowskie Góry Chemical Plant (periods of inverse proportionality are shaded *gray*)

Pines growing north of the zinc foundry (sites 6, 7, 8, 9, and 10)—a local source of pollution—showed reduced tree rings between 1960 and 2009 (Fig. 6). The greatest tree ring reductions occurred in 1970s, but reduced rings have been produced since the 1960s when the zinc foundry started to operate. In 1970–1990, the emissions of SO_2_ into the atmosphere from the plant were inversely proportional to the pine ring width at site 7, located relatively close to the zinc foundry (Fig. 8a). Due to local air pollution, pines growing near the foundry showed strongly reduced rings. The highest SO_2_ emissions were recorded in 1979. We found missing rings in 19 cores collected from trees growing at sites located close to the zinc foundry which demonstrates the extremely bad conditions for tree growth in that year. Additionally the winter of 1978/1979 was very harsh and also contributed to the reduced tree rings (Malik et al. 2009). For year 1979, a reduction was also found outside the zone influenced by pollution (at the reference site). When negative pointer years formed by climate conditions overlap with years of large harmful emission levels, tree ring reductions are relatively stronger (Vinš and Mrkva 1973; Jämbäck et al. 1999). To sum up, the strong tree ring reduction in 1979 was both due to climate conditions and air pollution.

### The Influence of Air Pollutants Emitted from the Tarnowskie Góry Chemical Plant on Pines Growth

Pines growing near the chemical plant—the second local polluter—produced reduced tree rings from 1950 until 1985. An increase in the production levels of the chemical plant is related to a decrease of tree ring width. This effect is clearly visible at site 11, located close to the chemical plant (Fig. 8b). An inverse ratio between the production volume of the chemical plant and tree ring width is clearly visible from 1955 until 1980. This means that at this time tree growth was limited by air pollution. Lots of missing rings were detected in trees growing near the chemical plant in the period 1963–1968 (Fig. 4b). It means that in the 1960s, tree growth was severely limited by air pollution from the chemical plant. Inhabitants of Czarna Huta—a village near the chemical plant—say that in this period, all plants growing within a 1-km radius of the chemical plant died. When the chemical plant authorities decide to hire a specialist responsible for introducing plants resistant to air pollution, it turned out that only the *Elaeagnus angustifolia* species was able to grow in the area. These bushes are still present around the ruins of the chemical plant (Malik et al. 2010).

An interesting thing is the large number of missing and reduced rings in the 1920s, which was determined at sites 2, 4, and 9 located near the chemical plant (Fig. 4a, b). The chemical plant started to operate in 1922, but there is not much information available for that first period of the plant’s history. The production of barium chloride started in 1926. It was produced in a muffle furnace, from which all the fumes were emitted directly into atmosphere without any filtration (Biernacki 1983). Relatively high amounts of tree ring reductions and a high number of missing rings in 1925–1930 prove the high air pollutant emissions from the chemical plant in the 1920s.

### The Relationship between Tree Ring Reductions Caused by Air Pollution and Adverse Effects on Human Health in the USID

**Fig. 9:**
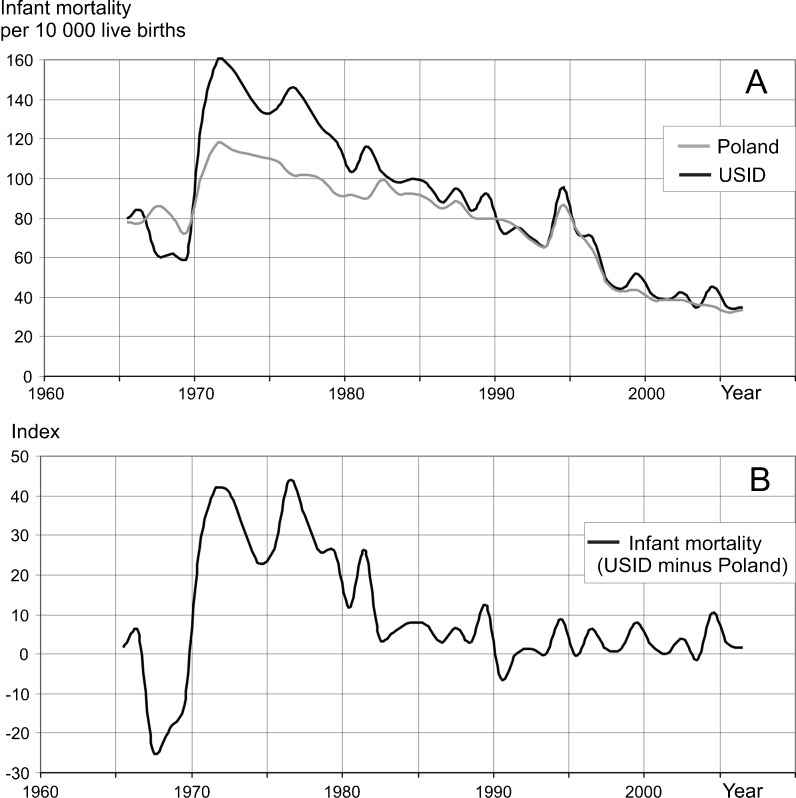
Relationship between infant mortality rates in Poland and the Upper Silesian Industrial District. **a** Variability of infant mortality rates in the USID and in Poland. **b** Infant mortality index (USID minus Poland)

Between 1970 and 1981, the infant mortality rate for newborn babies up to 4 weeks old in the Upper Silesian Industrial District was significantly higher than in the rest of Poland (Statistical Bulletins of Health Ministry, 1970–2009) (Fig. 9a, b).

**Fig. 10:**
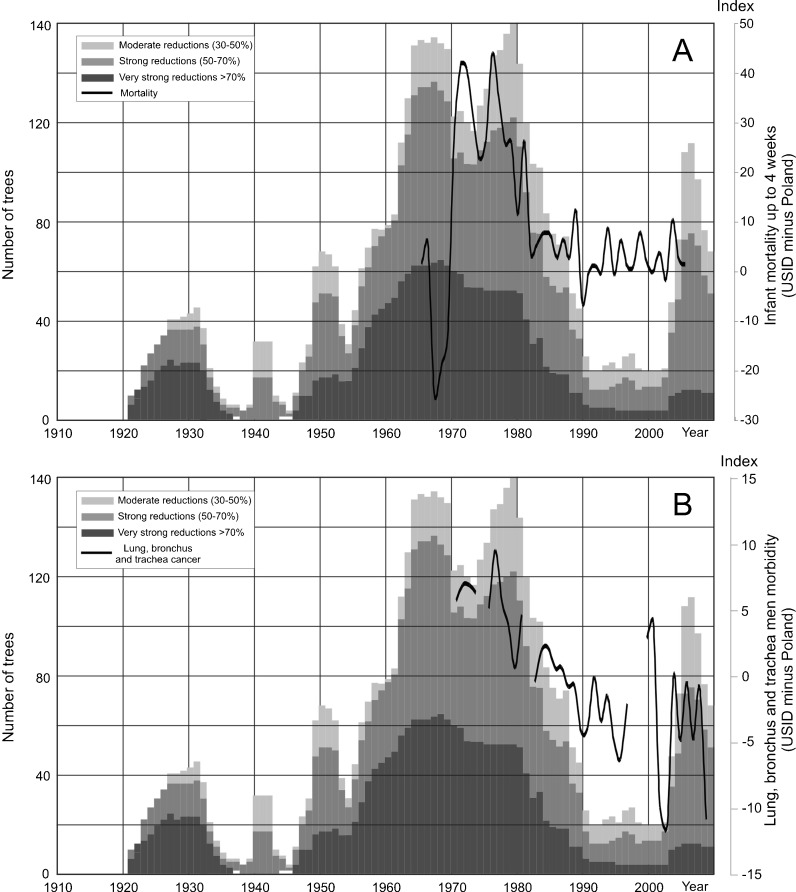
Relationship between the number of trees with reductions and adverse human health effects in the USID. **a** Relationship to infant mortality rates. **b** Relationship to lung, bronchial, and tracheal cancer morbidity rates among males

**Fig. 11:**
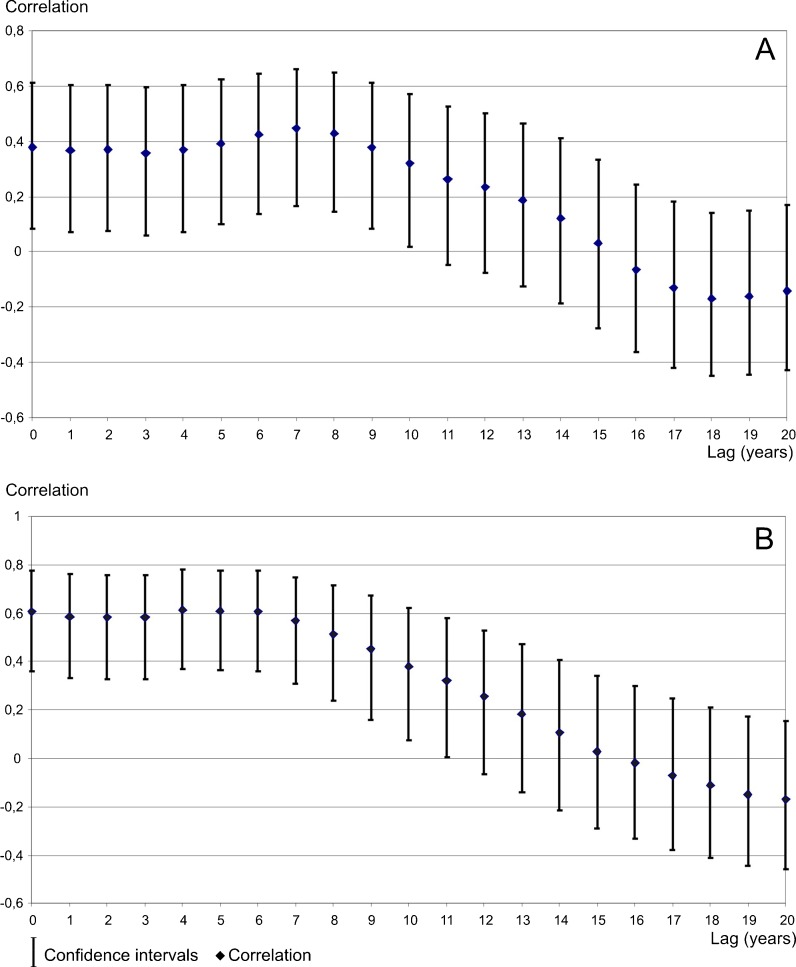
Correlation and confidence intervals for total tree ring reductions, infant mortality rates and lung, bronchial, and tracheal cancer morbidity rates among males. **a** Results for lagged detrended infant mortality rates. **b** Results for lagged detrended lung, bronchial, and tracheal cancer morbidity rates among males

The high amount of tree ring reductions in sampled pines affected by air pollution correlates with periods of high infant mortality rates in the district (Fig. 10a). The infant mortality rate in the USID and the rest of Poland has only been similar since 1981. The amount of tree ring reductions has also decreased since 1980 and reached a relatively low level in 1990. It is interesting that the increase in the number of tree ring reductions occurred earlier than the increase in infant mortality rates (Fig. 10a). This means that if the reaction of the trees to air pollution occurs before the infant mortality increase, it is possible that pines react more immediately and may have lower threshold values for pollutant content in the air. The calculation of the correlation between infant mortality and tree ring reduction shows the highest value of correlation coefficient (from 0.40 to 0.42) when a lag of 6–8 years is introduced between tree ring reduction and infant mortality, but there is also a correlation between data when there is no time lag (0.39) (Fig. 11a). It is convincing since there should be a time lag of at least 9 months due to the length of the pregnancy period when effects resulting from the exposure of the mother and fetus to air pollution can occur. As mentioned above, data shows longer lag periods in relation to high levels of air pollution. The increase of infant mortality over time (a 6–8-year delay) is, most probably, an effect of the long-term exposure to air pollution of women who were weakened even before pregnancy. There was a lag in the peak in infant mortality due to the cumulative effect of years of high air pollution emissions.

Up until 1987, lung, bronchial, and tracheal cancer morbidity rates among the male population in the Upper Silesian Industrial District were higher than in the rest of Poland (Statistical Bulletins of Health Ministry 1970–2009; Oncology Centre Report 2010).

**Fig. 12:**
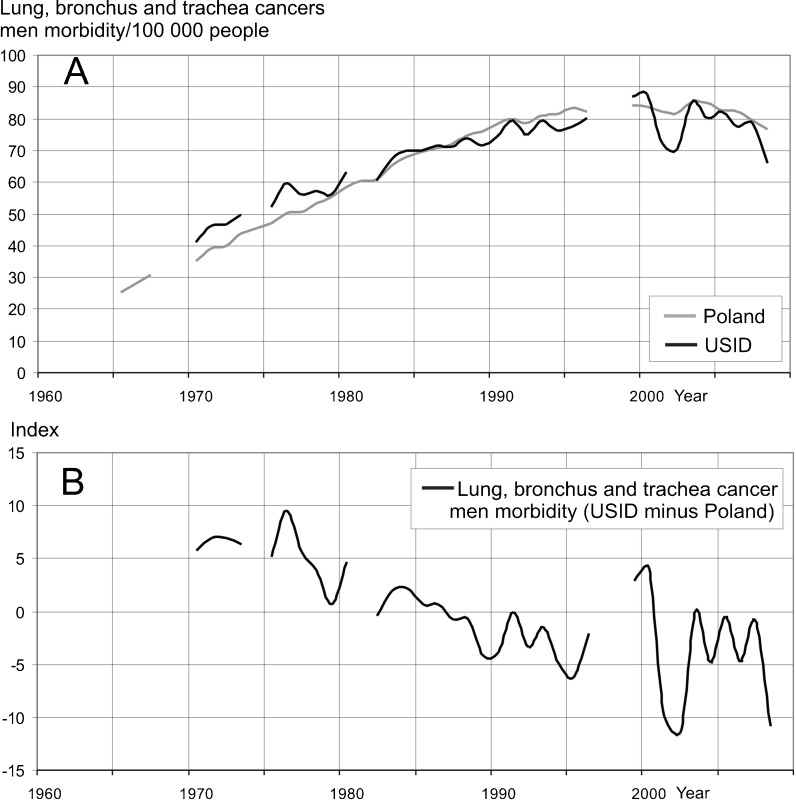
Relationship between lung, bronchial, and tracheal cancer morbidity rates among males in Poland and the Upper Silesian Industrial District. **a** Variability of lung, bronchial, and tracheal cancer morbidity rates for males in the USID and Poland. **b** Lung, bronchial, and tracheal cancer morbidity index for males (USID minus Poland)

After 1987, morbidity rates in the USID were lower (Fig. 12a, b). The number of cancer cases decreased since 1978, when it reached its highest level. It is difficult to say how it increased before that year because no data is available prior to 1970. The decrease in cancer morbidity rates is slower than the decrease in the infant mortality rate and the number of tree ring reductions (Fig. 10b). This seems normal because cancer develops over a longer period and should be more lagged in relation to air pollution than infant mortality and the increase in tree ring reductions. The calculation of the correlation of lung, bronchial, and tracheal cancer rates related to tree ring reductions showed the highest correlation values (around 0.6) when human health data is lagged 0–6 years (Fig. 11b). In the case of cancer, this lag could even extend to 20–30 years (Mossman and Gee 1989; Doll et al. 2004) due to the latency period. On the other hand, the strongest correlation between the number of tree ring reductions and morbidity of cancer of the lung, bronchus, and trachea was observed when there was a delay of up to 6 years to cancer occurrence. The exact biological mechanism of the effect of air pollution on human health, including the potential delay in the occurrence of health outcomes, is unknown. Maybe an extremely high air pollution level produced an accelerated development of cancer. Also, previous air pollution emissions (from the period before the 1960s–1980s) can be responsible for relatively high correlations in the case where the cancer data has a lag of 0–6 years following the number of tree rings data. The value of correlation coefficient calculated for cancer morbidity rates among males is higher than the correlation for infant mortality (Fig. 11a, b).

The relatively high correlation for cancer morbidity rates is possibly due to the type of group selected for studies. The male population had greater exposure to air pollution—they performed hard physical work at heavy industrial plants in the USID, so they were endangered because of the inhalation of highly polluted air.

### Is it Possible to Use Tree Ring Reductions as an Indicator of Future Adverse Effects on Human Health?

The preliminary results of this study show that it may be possible to create the basis for a model, which could be developed and used as a tool to forecast the adverse effects of air pollution on human health. Of course, the correlation model presented is very simple and makes many assumptions (such as the constant lag between the reaction in trees and people over time). Additionally, chemical analyses of tree rings and soil should be helpful in developing the method of forecasting. The creation of reliable forecasting model needs further studies. The model should be based on the measurements of ring reductions in selected tree species which are recognized to be the best indicators of air pollution. Studies carried out in Japan confirmed that adverse human health effects can be linked to the occurrence of ring reductions (Kagamimori et al. 1990). If applied to developed countries, the model may support instrumental monitoring systems (networks of measurement stations), providing information about the combined effects of pollutants and allowing health consequences in populations exposed to air pollution to be forecast.

A health risk forecasting model would take into account the following: the relationship between tree reaction and the occurrence of negative health symptoms among humans. The development of the model seems to be possible thanks to the identified latency period, which varies depending on the type of health effect.

The opportunity to create a biological network that would provide advance warning about health hazards seems to be significant for developing countries, where the local economy mainly relies upon conventional sources of energy (coal, wood, and gas) and where the automatic stations for monitoring air pollution control are not used for various reasons (e.g., India, China, Brazil, Russia, etc.).

Nevertheless, the use of tree ring reductions as an indicator of adverse effects on human health needs more study sites and epidemiological data to compare human mortality and morbidity rates and tree ring reductions. Cooperation between forest ecologists, environmental health, soil, and air pollution specialists, and dendrochronologists is therefore necessary.

## Conclusions

1. Pines growing north of the Upper Silesian Industrial District (USID) formed reduced rings in the period 1950–1990. The reductions occurred as an effect of particularly high levels of air pollution emissions. The level of emissions was determined indirectly using data on coal production and the production of electricity and steel in the USID. An especially great negative impact of pollution on pine growth was recorded in the period 1964–1981 when many missing rings were found in the pines studied. At the same time, pines growing 60 km west from the USID do not record large ring reductions proving that the phenomenon is of a regional nature.
2. The ring reductions in pines growing closest to the Miasteczko Śląskie Zinc Foundry and Tarnowskie Góry Chemical Plants occurred simultaneously with high levels of emission of sulfur dioxide from the foundry and simultaneously with an increase in the volume of production in the chemical plant.
3. An increase in infant mortality and in morbidity of cancer of the lung, bronchus, and trachea occurred several years after the increase in the number of tree ring reductions observed in the USID. Infant mortality shows a lag of 6–8 years in relation to tree ring reductions, a correction also occurs when there is a lag in the data, which is convincing due to the length of pregnancy period. The lag of 6–8 years effect seems to be the result of long-term exposure of women to air pollution even before pregnancy. The adverse health effects were cumulative and infant mortality increased with the time lag. There is a time lag of up to 6 years in the case of the relationship between tree ring data and the morbidity of cancer of the lung, bronchus, and trachea. The value of correlation coefficient, calculated for cancer morbidity rates among males, is higher than the correlation for infant mortality. Probably the male population had greater exposure to air pollution—they performed hard physical work at heavy industrial plants in the USID, so they were endangered because of the inhalation of highly polluted air.
4. Pines produce reduced rings immediately after an increase in air pollution. Adverse human health effects show a lag of several years after an increase in air pollution. Therefore, it seems to be possible to use tree ring reductions as an indicator of future adverse effects on human health especially in countries where automatic stations for monitoring air pollution are not available. Using tree ring reductions as an indicator has some limitations, e.g., the different sensitivities of tree species to air pollution and the different latency periods depending upon the type of human health effect. Therefore, further studies are required to specify the potential to apply tree ring reductions as an indicator of adverse effects on human health.

## References

[CR1] Absalon D, Ślesak B (2010). The effects of changes in cadmium and lead air pollution on cancer incidence in children. Science of the Total Environment.

[CR2] Ashby WC, Fritts HC (1972). Tree growth, air pollution, and climate near LaPorte, Indiana. Bulletin of the American Meteorological Society.

[CR3] Bell ML, Ebisu K, Belanger K (2007). Ambient air pollution and low birth weight in Connecticut and Massachusetts. Environmental Health Perspectives.

[CR4] Biernacki, W. (1983). *60 lat Zakładów Chemicznych “Tarnowskie Góry”.* Tarnowskie Góry: Zakłady Chemiczne „Tarnowskie Góry”. Statistical Bulletins of Polish Health Ministry, 1970–2009, Warszawa: Centrum Systemów Informacyjnych Ochrony Zdrowia.

[CR5] Bojanowski, S. (2008). *Huta Cynku „Miasteczko Śląskie” S.A. wczoraj i dziś.* Miasteczko Śląskie: Huta Cynku „Miasteczko Śląskie”.

[CR6] Cohen AJ, Anderson HR, Ostro B, Pandey KD, Krzyzanowski M, Kuenzli N (2005). The global burden of disease due to outdoor air pollution. Journal of Toxicology and Environmental Health.

[CR7] Cook, E. R., & Holmes, R. L. (1999). *User manual for Program ARSTAN.* Arizona, Tucson: Laboratory of Tree-Ring Research, University of Arizona.

[CR8] Danek M (2007). The influence of industry on Scots pine stands in the south-eastern part of the Silesia–Kraków Upland (Poland) on the basis of dendrochronological analysis. Water, Air, & Soil Pollution.

[CR9] Doll R, Peto R, Boreham J, Sutherland I (2004). Mortality in relation to smoking: 50 years’ observations on male British doctors. British Medical Journal.

[CR10] Elling W, Dittmar Ch, Pfaffelmoser K, Rotzer T (2009). Dendroecological assessment of the complex causes of decline and recovery of the growth of silver fir (*Abies alba* Mill.) in Southern Germany. Forest Ecology and Management.

[CR11] Farrar JF, Relton J, Rutter AJ (1977). Sulphur dioxide and the scarcity of *Pinus sylvestris* in the industrial Pennines. Environmental Pollution.

[CR12] Gray SC, Edwards SE, Miranda ML (2010). Assessing exposure metrics for PM and birth weight models. Journal of Exposure Science & Environmental Epidemiology.

[CR13] Hoek G, Brunekreef B, Goldbohm S, Fischer P, Brandt PA (2002). Association between mortality and indicators of traffic-related air pollution in the Netherlands: a cohort study. Lancet.

[CR14] Ivshin AP, Shiytov SG (1995). The assessment of subtundra forest degradation by dendrochronological methods in the Norilsk industrial area. Dendrochronologia.

[CR15] Jämbäck J, Heikkinen O, Tuovinen M, Autio J (1999). The effect of air-borne pollutants on the growth of *Pinus sylvestris* in the City of Oulu, Finland. Fennia.

[CR16] Juknys R, Vencloviene J, Stravinskiene V, Augustaitis A, Bartkevicius E (2003). Scots pine (*Pinus sylvestris* L.) growth and condition in a polluted environment: from decline to recovery. Environmental Pollution.

[CR17] Kagamimori S, Katoh T, Naruse Y, Kakiuchi H, Matsubara I, Kasuya M (1990). An ecological study on air pollution: changes in annual ring growth of the Japanese cedar and prevalence of respiratory symptoms in schoolchildren in Japanese rural districts. Environmental Research.

[CR18] Kennedy-Sutherland E, Martin B (1990). Growth response of *Pseudotsuga menziesii* to air pollution from cooper smelting. Canadian Journal of Forest Research.

[CR19] Kontic R, Winkler-Seifert A, Kairiukstis A, Nilsson S, Straszak A (1987). Comparative studies on the annual ring pattern and crown conditions of conifers. Forest decline and reproduction.

[CR20] Kowalska M, Hubicki L, Zejda JE, Osródka L, Krajny E, Wojtylak M (2007). Effect of ambient air pollution on daily mortality in Katowice Conurbation, Poland. Polish Journal of Environmental Study.

[CR21] Kowalska M, Zejda JE, Skrzypek M (2010). Short-term effect of ambient air pollution on daily mortality. Polish Journal of Environmental Study.

[CR22] Krąpiec M, Szychowska-Krąpiec E (2001). Tree-ring estimation of the effect of industrial pollution on pine (*Pinus sylvestris*) and fir (*Abies alba*) in the Ojców National Park (Southern Poland). Nature Conservation.

[CR23] Malik I, Danek M, Danek T, Krąpiec M, Wistuba M (2009). Zanieczyszczenie atmosfery przez zakłady przemysłowe położone w północnej części Wyżyny Śląskiej zapisane w przyrostach rocznych sosny zwyczajnej. Czasopismo Geograficzne.

[CR24] Malik I, Danek M, Krąpiec M (2010). Air pollution recorded in Scots pine growing near a chemical plant, preliminary results and perspective (Upper Silesia, southern Poland). TRACE—Tree Rings in Archaelogy Climatology and Ecology.

[CR25] Marchwinska-Wyrwał E, Dziubanek G, Skrzypek M, Hajok I (2010). Study of the health effects of long-term exposure to cadmium and lead in a region of Poland. International Journal of Environmental Health Research.

[CR26] Maroziene L, Grazuleviciene R (2002). Maternal exposure to low-level air pollution and pregnancy outcomes: a population-based study. Environmental Health.

[CR27] Mossman BT, Gee JB (1989). Asbestos-related diseases. The New England Journal of Medicine.

[CR28] Nash TH, Fritts HC, Stokes MA (1975). A technique for examining nonclimatic variation in widths of annual tree rings with special reference to air pollution. Tree-Ring Bulletin.

[CR29] Nöjd P, Mikkola K, Saranpää P (1996). History of forest damage in Monchegorsk, Kola; a retrospective analysis based on tree rings. Canadian Journal of Forest Research.

[CR30] Oleksyn J (1988). High growth of different European Scots pine provenances in a heavy polluted and control environment. Environmental Pollution.

[CR31] Oncology Centre Report (2010). Webpage founded by Health Ministry from National Cancer Prevention Program. http://85.128.14.124/krn/.

[CR32] Pope CA (2007). Mortality effects of longer term exposure to fine particulate air pollution: review of recent epidemiological evidence. Inhalation Toxicology.

[CR33] Schweingruber, F. H., Kontic, R., Niederer, M., Nippel, C. A., & Winkler-Seifert, A. (1985). Diagnosis and distribution of conifer decay in the Swiss Rhone Valley a dendrochronological study. In: H. Turner, W. Tranquillini (Eds.) *Establishment and tending of subalpine forest* (pp. 189–192). Berno: Swiss Federal Institute of Forestry Research.

[CR34] Schweingruber FH, Eckstein D, Serre-Bachet F, Bräker OU (1990). Identification, presentation and interpretation of event years and pointer years in dendrochronology. Dendrochronologia.

[CR35] Statistical Yearbooks of Katowice/Silesian Voivodeship, 1958–2009, Katowice: Wojewódzki Urząd Statystyczny w Katowicach.

[CR36] Stoeckhardt JA (1871). Untersuchungen uber die schadliche Einwirkung des Hutten- und Steinkohlenrauches auf das Wachsthum der Pflanzen, insbesondere der Fichte und Tanne. Tharandter forstliches Jahrbuch.

[CR37] Szychowska-Krapiec E, Wiśniewski Z (1996). Zastosowanie analizy przyrostów rocznych sosny zwyczajnej (Pinus silvestris) do oceny wpływu zanieczyszczeń przemysłowych na przykładzie zakładów chemicznych "Police" (woj. szczecińskie). Kwartalnik Akademii Górniczo-Hutniczej, Geologia.

[CR38] The European Health Report 2009. World Health Organization. (http://www.euro.who.int/__data/assets/pdf_file/0009/82386/E93103.pdf).

[CR39] Thompson MA (1981). Tree ring and air pollution: a case study of *Pinus monophylla* growing in east-central Nevada. Environmental Pollution.

[CR40] Vinš B, Mrkva R (1973). The diameter increment losses of pine stands as a result of injurious emissions. Acta Universatis Agriculturae.

[CR41] Walanus, A. (2002). *Program DenMy i DenDruk.* Kraków.

[CR42] Wilczyński S, Skrzyszewski J (2002). The climatic signal in tree-rings of Scots pine (*Pinus sylvestris* L.) from foot-hills of the Sudetic Mountains (southern Poland). Forstwissenschaftliches Centralblatt.

[CR43] Zejda, J. E. (2000). Health effect of ambient air pollution—the magnitude of risk and current hazard in Poland In K. Janicki, W. Klimza, J. Szewczyk (Ed.), *Environment and health.* Czestochowa: Cmyk-Art.

[CR44] Zielski A (1996). Wpływ temperatury i opadów na szerokość słojów rocznych drewna u sosny zwyczajnej (Pinus sylvestris L) w rejonie Torunia. Sylwan.

